# Supplementary material for: “Smart Extraction Chain” with Green Solvents: Extraction of Bioactive Compounds from Picea abies Bark Waste for Pharmaceutical, Nutraceutical and Cosmetic Uses
Source: Molecules. 2022 Oct 9;27(19):6719. doi: 10.3390/molecules27196719 (PMC9571752; doi:10.3390/molecules27196719)
Supplement: Supplementary file 1 [file molecules-27-06719-s001.zip › molecules-1923746-supplementary.pdf]

"Smart extraction chains" with green solvents: extraction of bioactive compounds from *Picea abies* bark waste for pharmaceutical, nutraceutical and cosmetic uses

Table S1. Yields of extraction of *P. abies* samples. SCO2: supercritical fluid extract; R: residual bark material after SCO2; B: bark material; U: ultrasound-assisted extraction; MW: microwave assisted extraction; M: maceration w: water; we: water/ethanol 50%; e: ethanol

**Sample**	Abietane-type diterpenoids	Piceasides and derivates	Flavonoids and phenolics
BU_w	mg/kg of bark	mg/kg of bark	mg/kg of bark
BU_we	8098.56	15.09	228.33
BU_e	171.92	679.93	1678.18
RU_w	328.38	3894.95	4029.15
RU_we	333.62	1400.91	1640.29
RU_e	88.27	206.46	399.91
BMW_w	1600.72	835.42	1966.82
BMW_we	3827.89	441.31	1101.24
BMW_e	41.79	1170.52	1992.66
RMW_w	454.13	5676.38	5752.89
RMW_we	506.33	2921.85	3665.08
RMW_e	101.84	209.04	400.63
BM_w	1369.07	1519.35	1948.56
BM_we	2960.8	410.44	872.86
BM_e	32.12	781.81	1831.37
RM_w	357.63	4397.66	4594.13
RM_we	365.99	1759.06	2130.7
RM_e	36.33	188.29	235.36
SCO2	935.1	559.04	842.24

Table S2. Total phenolic, flavonoid content, and free radical scavenging abilities of the tested extracts

**Sample**	**TPC (mg GAE/g)**	**TFC (mg RE/g)**	**DPPH (mg TE/g)**	**ABTS (mg TE/g)**
BU_w	78.85±1.52	1.16±0.20	172.15±3.25	486.39±15.12
BU_we	118.66±0.45	3.83±0.16	378.38±11.63	754.08±7.78
BU_e	90.62±0.86	4.21±0.19	248.82±7.02	533.37±15.67
RU_w	38.87±2.04	1.59±0.04	48.71±0.87	134.48±1.23
RU_we	122.08±2.38	3.33±0.22	58.10±0.14	151.99±0.20
RU_e	123.79±0.35	7.38±0.18	600.91±2.29	1053.89±14.88

BMW_w	81.81±0.99	3.88±0.19	201.36±5.05	379.79±6.50
BMW_we	118.63±1.47	5.42±0.14	454.17±6.33	840.11±5.72
BMW_e	95.78±1.74	5.62±0.36	239.20±1.57	507.78±6.15
RMW_w	110.79±1.71	2.03±0.27	371.62±0.50	640.69±7.79
RMW_we	124.48±0.61	4.25±0.16	521.13±2.72	984.87±5.05
RMW_e	121.80±1.84	7.60±0.66	491.07±6.99	941.34±9.90
BM_w	62.15±2.26	1.08±0.16	105.99±1.33	252.39±1.41
BM_we	117.54±0.47	4.00±0.20	370.91±4.70	769.82±3.54
BM_e	67.36±1.41	4.46±0.30	106.29±1.51	305.25±2.13
RM_w	100.97±1.89	1.30±0.08	285.08±4.11	492.11±3.73
RM_we	122.61±0.87	3.57±0.20	496.01±7.36	891.03±4.40
RM_e	125.82±0.83	7.85±0.17	604.36±5.98	1225.75±2.62
SCO2	24.71±0.33	0.31±0.02	17.03±0.33	54.48±0.39

*Values are reported as mean±SD of three parallel measurements. TPC: Total phenolic content; TFC: Total flavonoid content; GAE: Gallic acid equivalent; RE: Rutin equivalent; TE: Trolox equivalent.

Table S3. Reducing power (CUPRAC and FRAP), metal chelating (MCA) and total antioxidant capacity (by phosphomolybdenum assay (PBD)) of the tested extracts

Sample	CUPRAC (mg TE/g)	FRAP (mg TE/g)	MCA (mg EDTAE/g)	PBD (mmol TE/g)
BU_w	202.53±3.22	174.24±0.25	6.47±0.37	1.56±0.03
BU_we	529.92±5.62	333.52±6.03	7.60±0.68	1.98±0.19
BU_e	383.09±6.49	250.64±1.38	5.40±0.67	1.94±0.15
RU_w	101.73±0.15	83.82±0.93	Na	1.50±0.15
RU_we	326.32±4.16	185.96±2.09	2.64±0.26	2.03±0.06
RU_e	649.49±13.96	482.30±8.33	4.41±0.34	2.26±0.04
BMW_w	306.12±4.53	208.75±1.77	9.91±0.89	1.09±0.01
BMW_we	512.62±10.40	354.40±3.86	5.89±0.67	1.96±0.13
BMW_e	360.43±7.15	232.88±3.11	4.75±0.19	1.59±0.07
RMW_w	415.47±12.62	295.29±3.97	5.36±0.57	1.69±0.01
RMW_we	702.55±4.98	422.43±5.24	4.64±0.09	2.35±0.16
RMW_e	554.59±3.46	380.52±6.97	4.00±0.52	1.97±0.01
BM_w	160.91±4.09	120.85±0.83	7.17±0.07	0.90±0.02
BM_we	534.23±0.96	333.93±3.44	8.70±0.28	1.84±0.10
BM_e	198.25±1.03	134.28±0.27	2.66±0.06	2.26±0.26
RM_w	347.39±6.34	258.22±2.68	3.72±0.13	1.56±0.04

RM_we	629.65±2.44	401.19±3.13	3.36±0.52	2.52±0.01
RM_e	747.00±14.78	496.95±3.01	4.54±0.83	2.95±0.07
SCO2	55.43±0.09	22.82±2.08	4.22±0.20	2.10±0.01

*Values are reported as mean±SD of three parallel measurements. TE: Trolox equivalent; EDTAE: EDTA equivalent; Na: not active

MS spectra of most significant *Picea abies* bark extracts

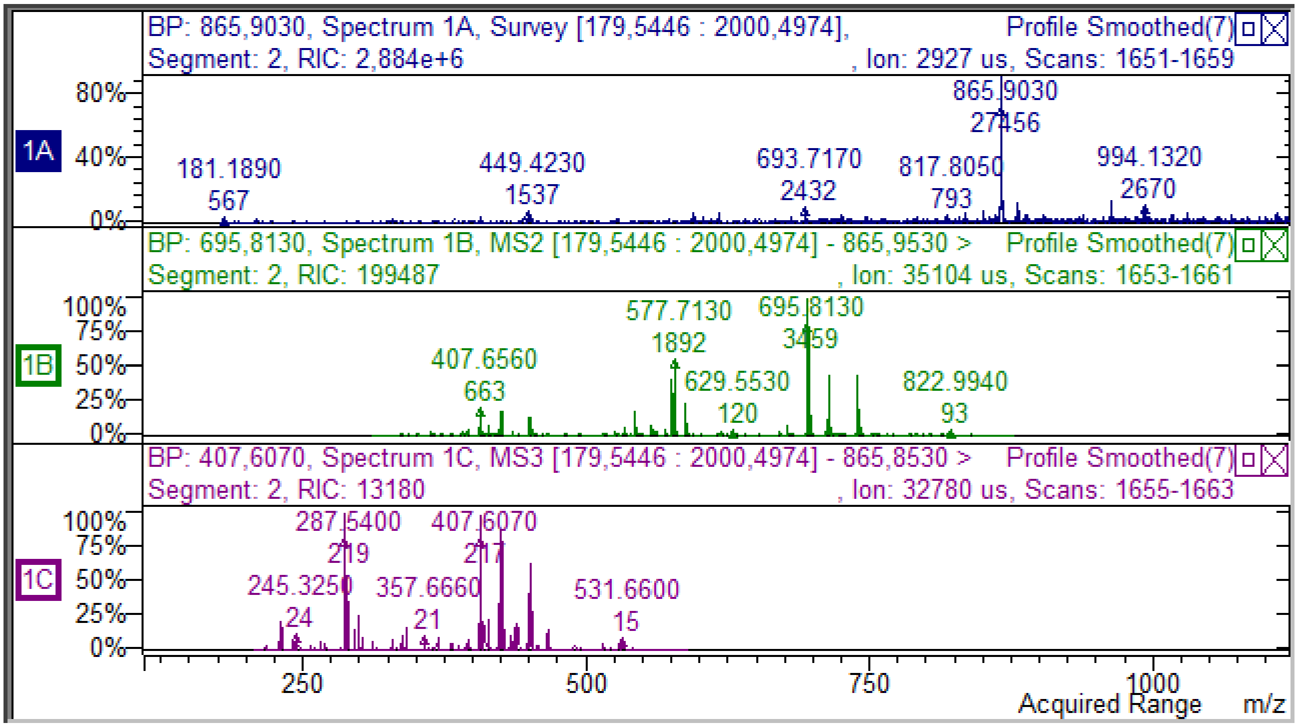

Procyanidin trimer B

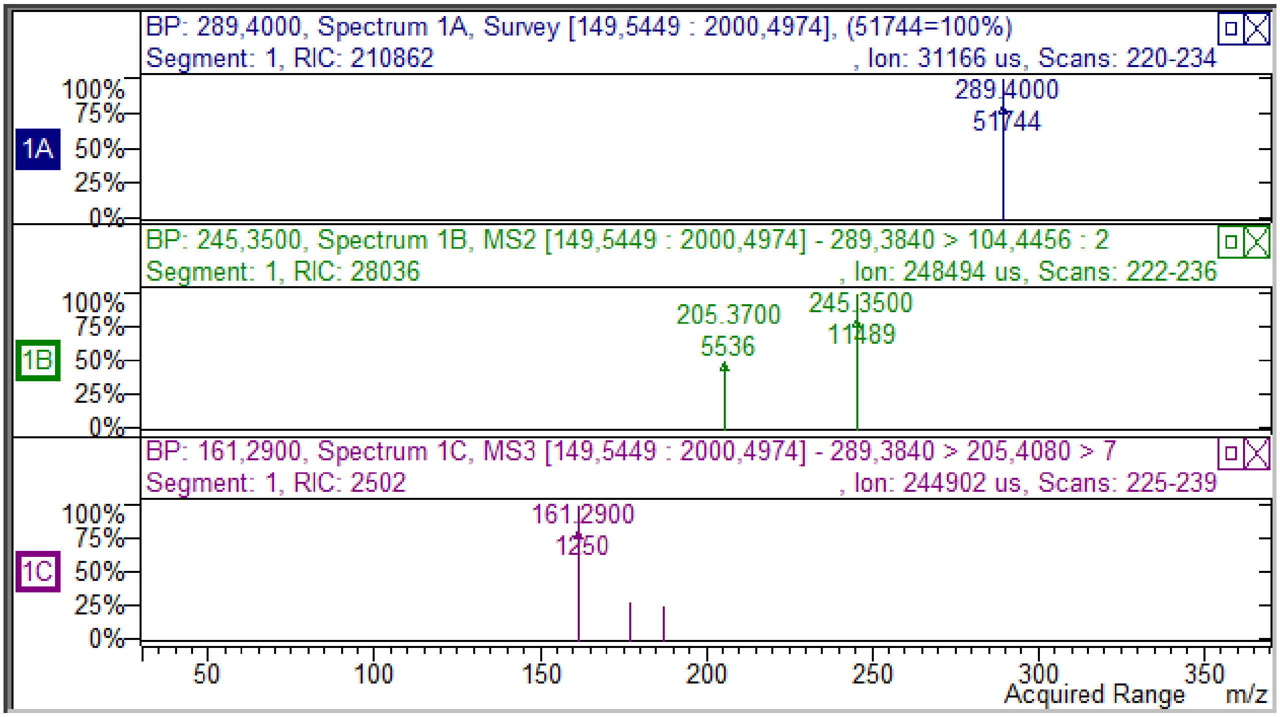

(epi)-Catechin

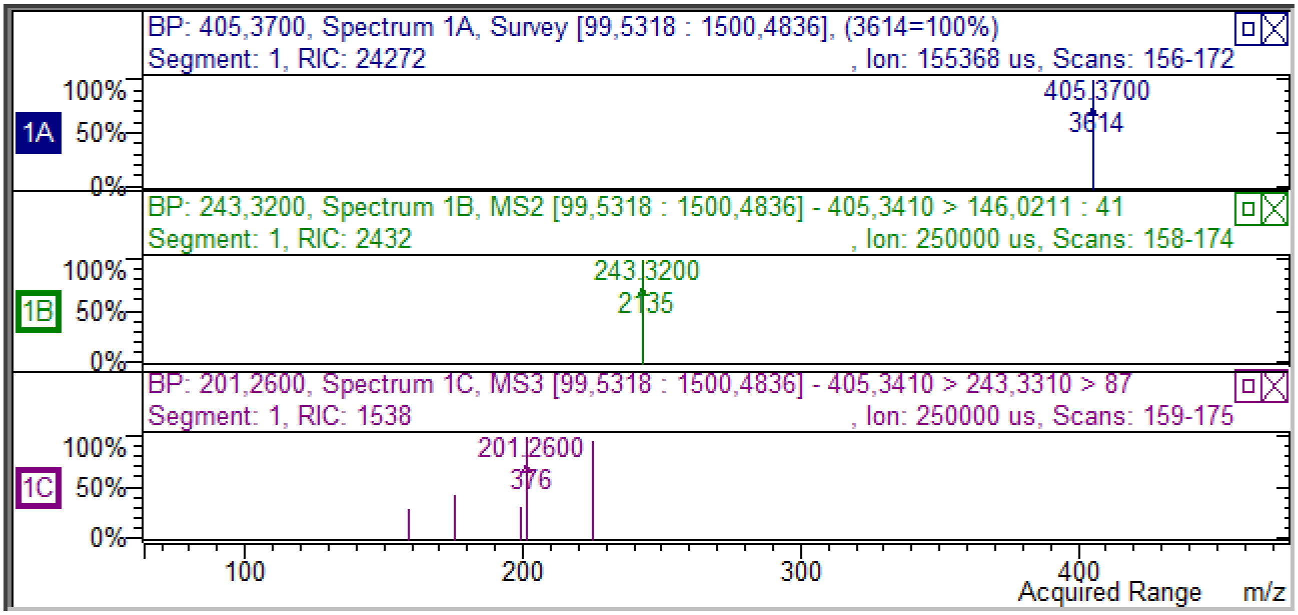

Trans-astringin

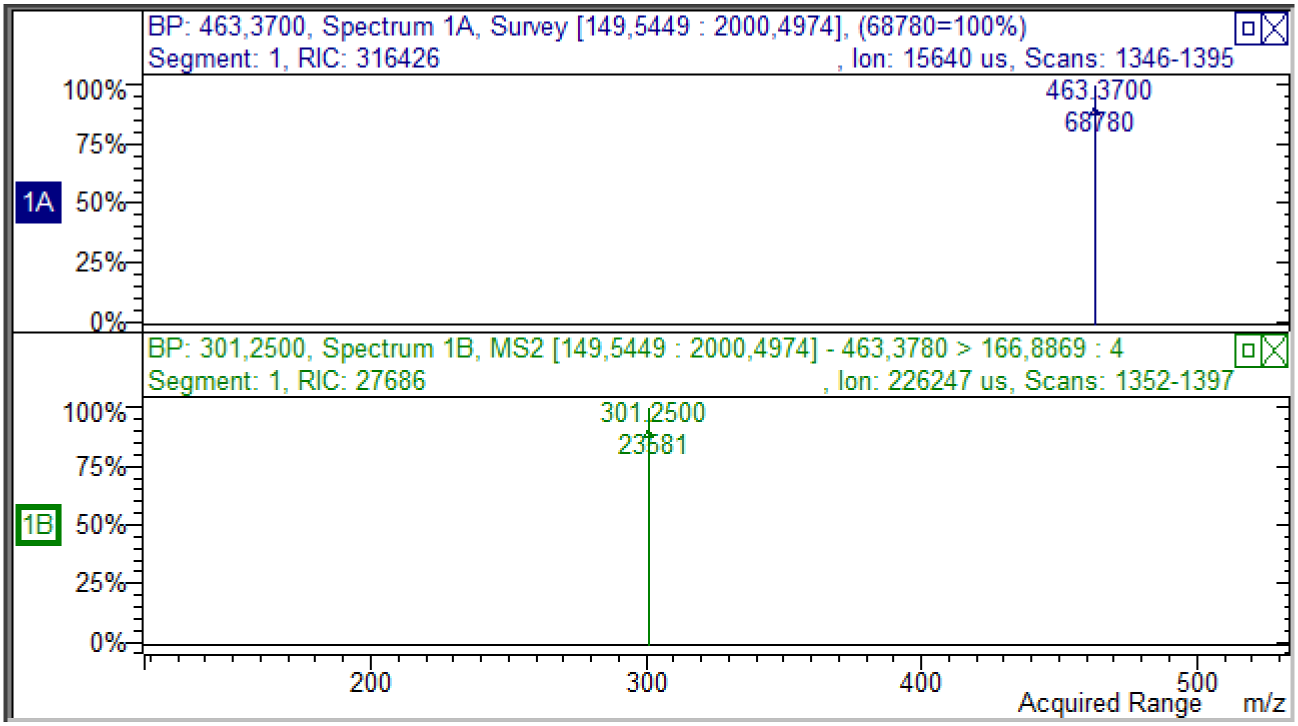

Ellagic acid hexoside

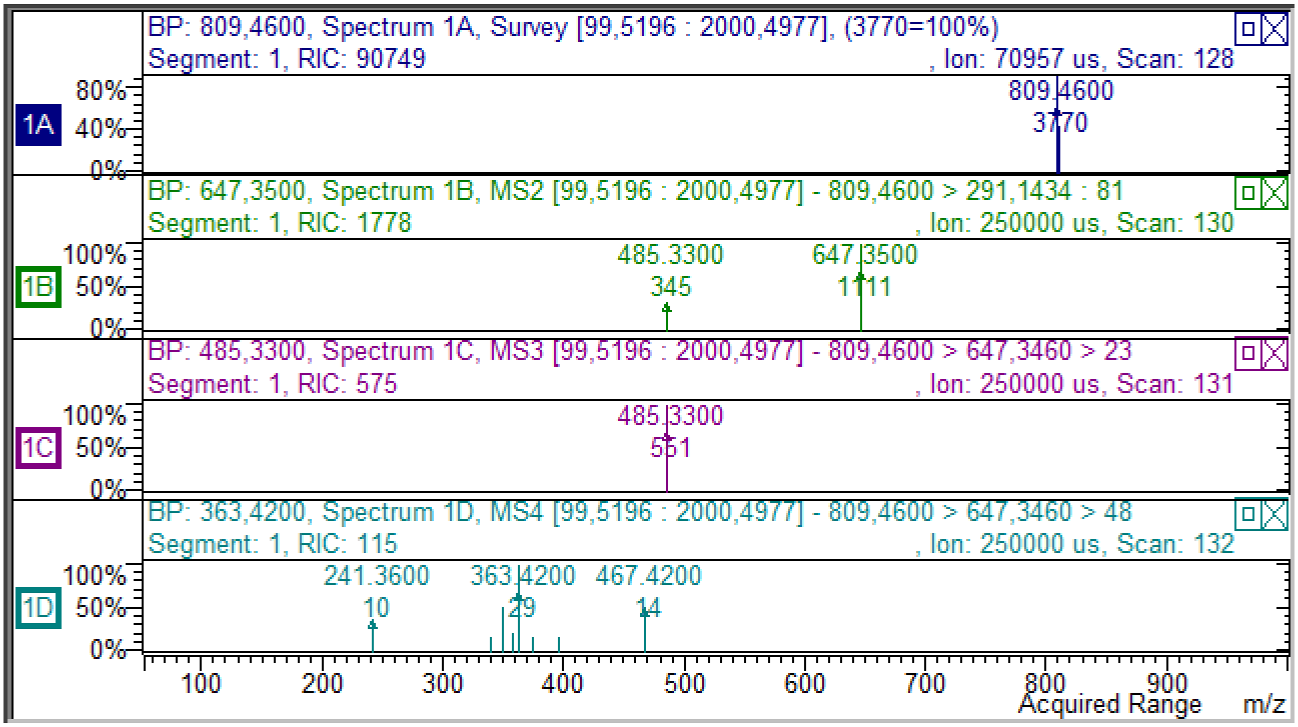

Piceaside A/B

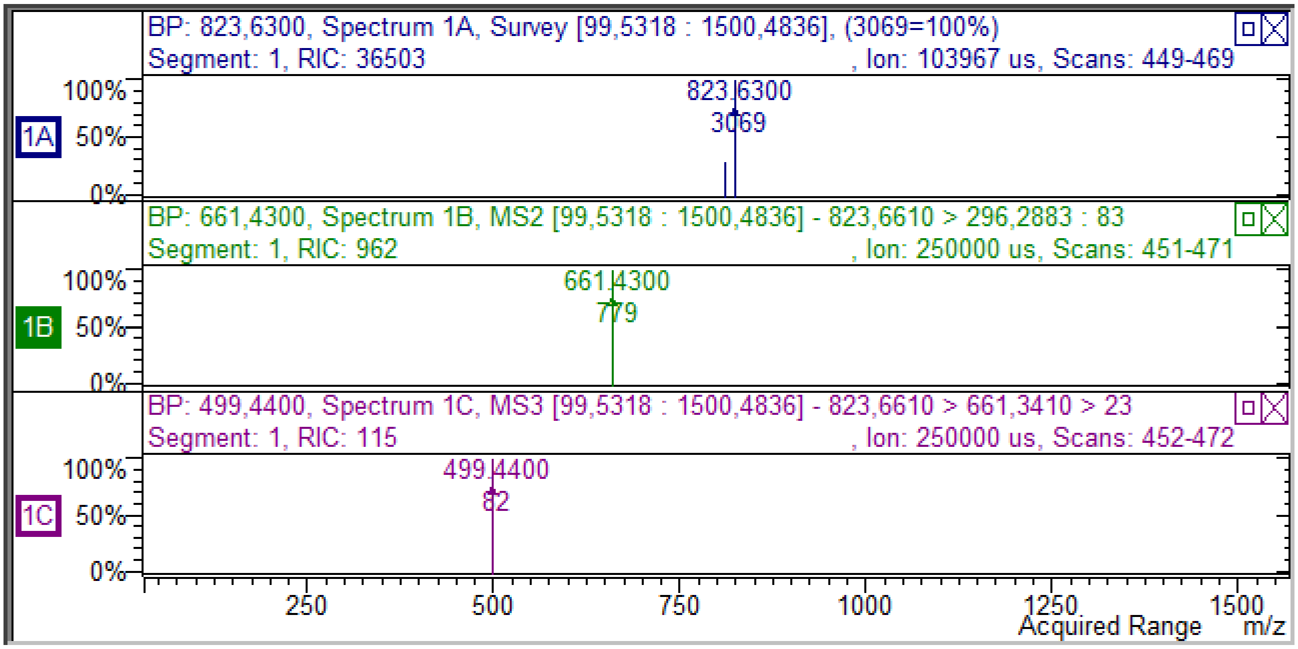

Piceaside C/D

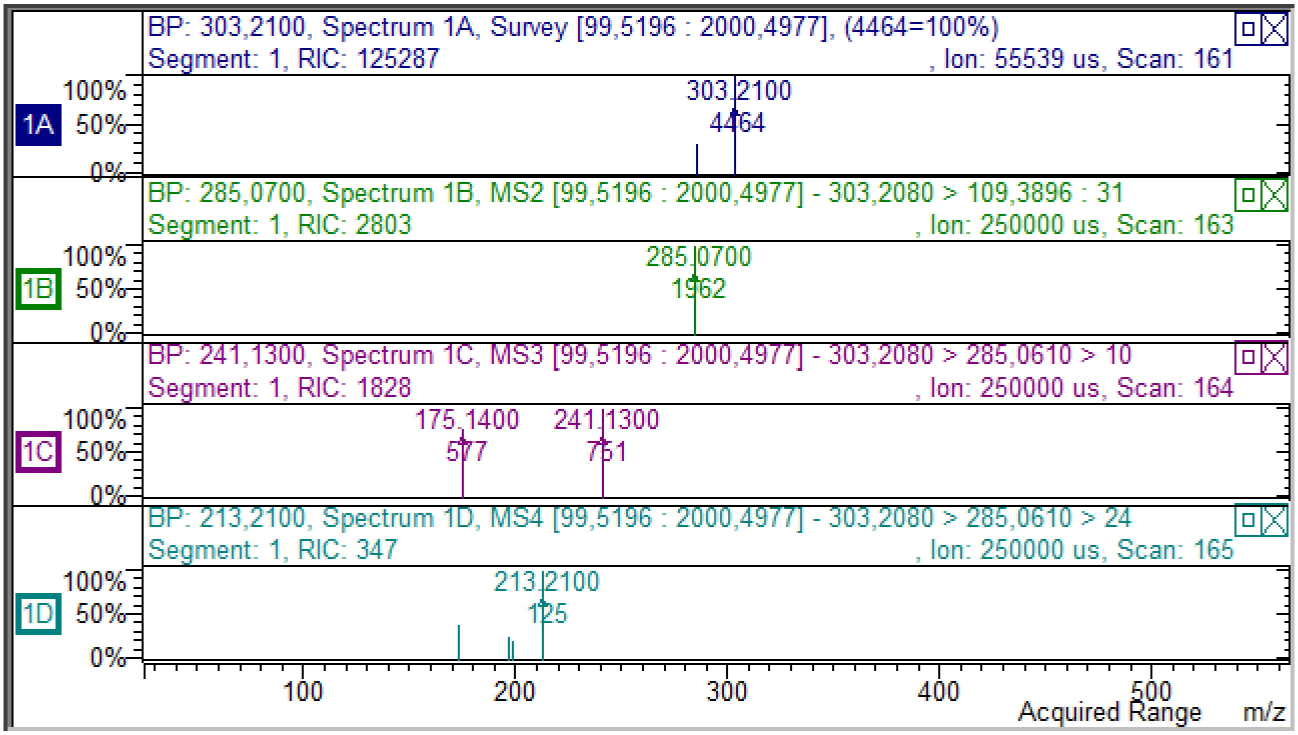

Taxifolin

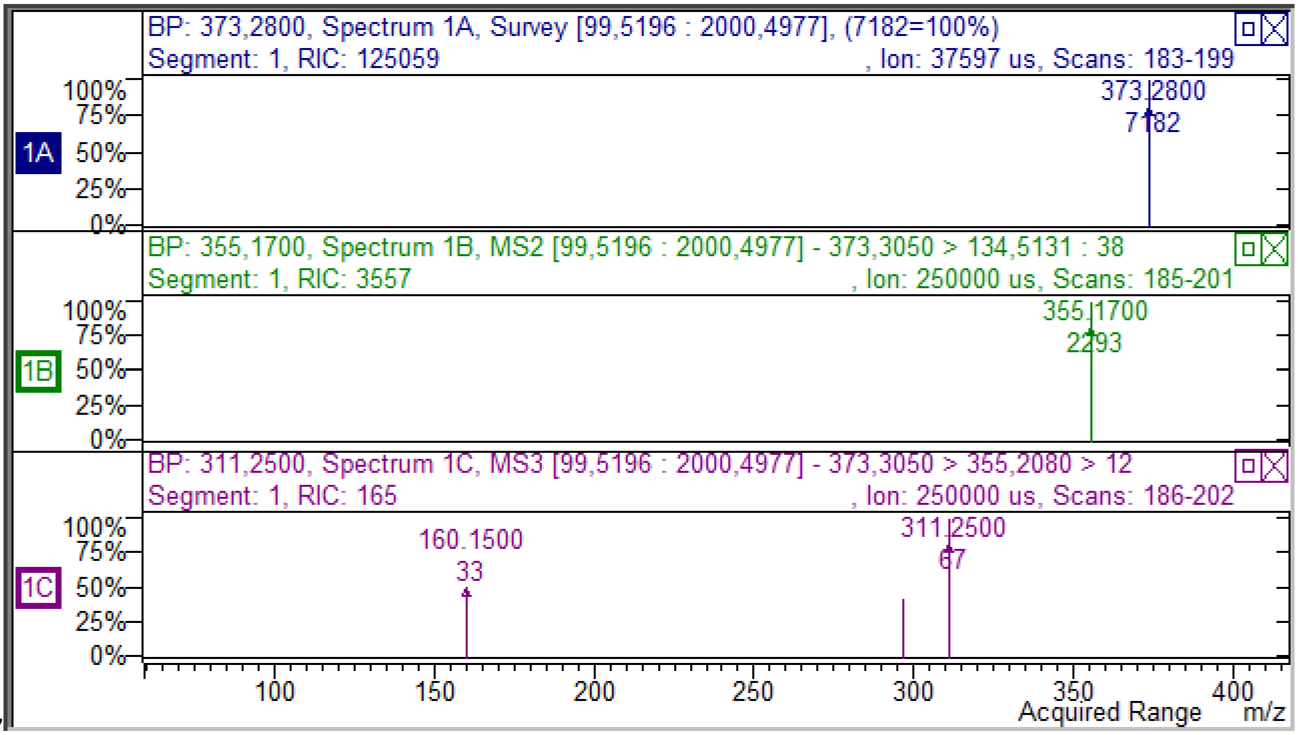

7-OH matairesinol

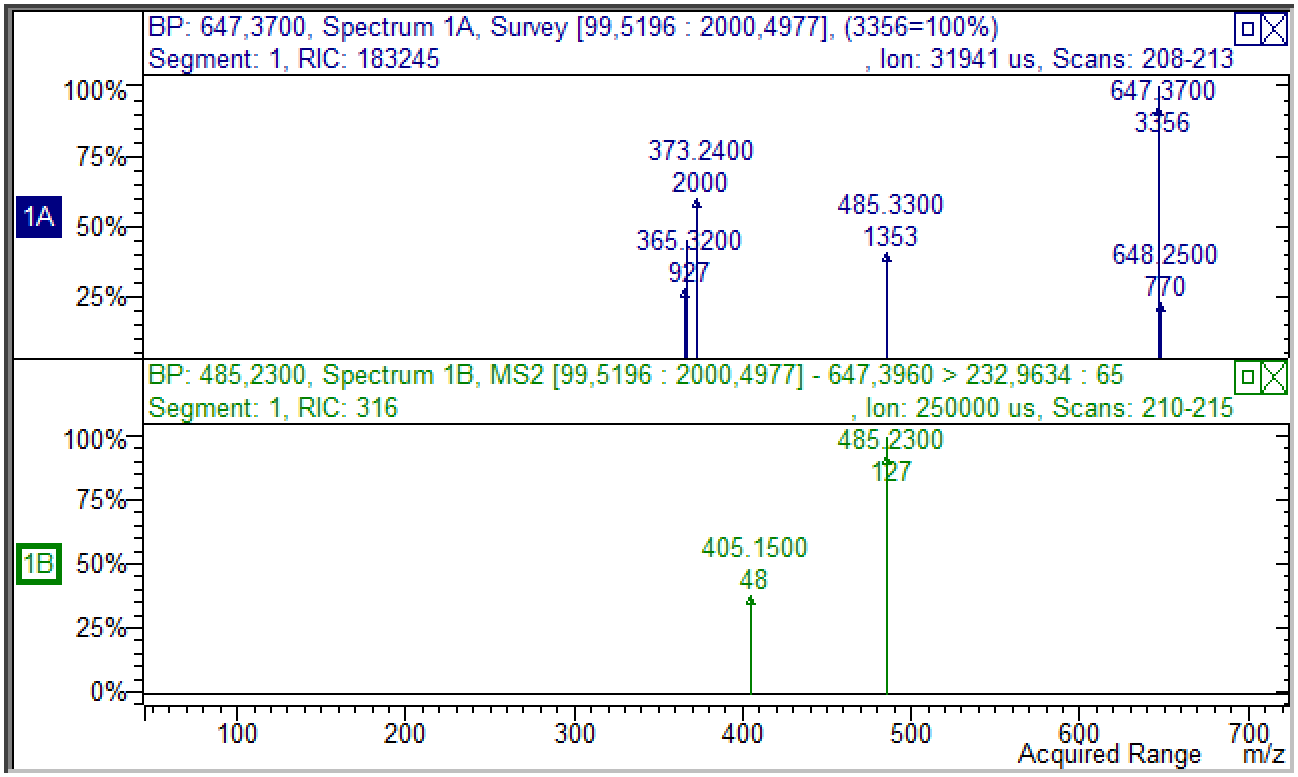

Piceatannol derivative

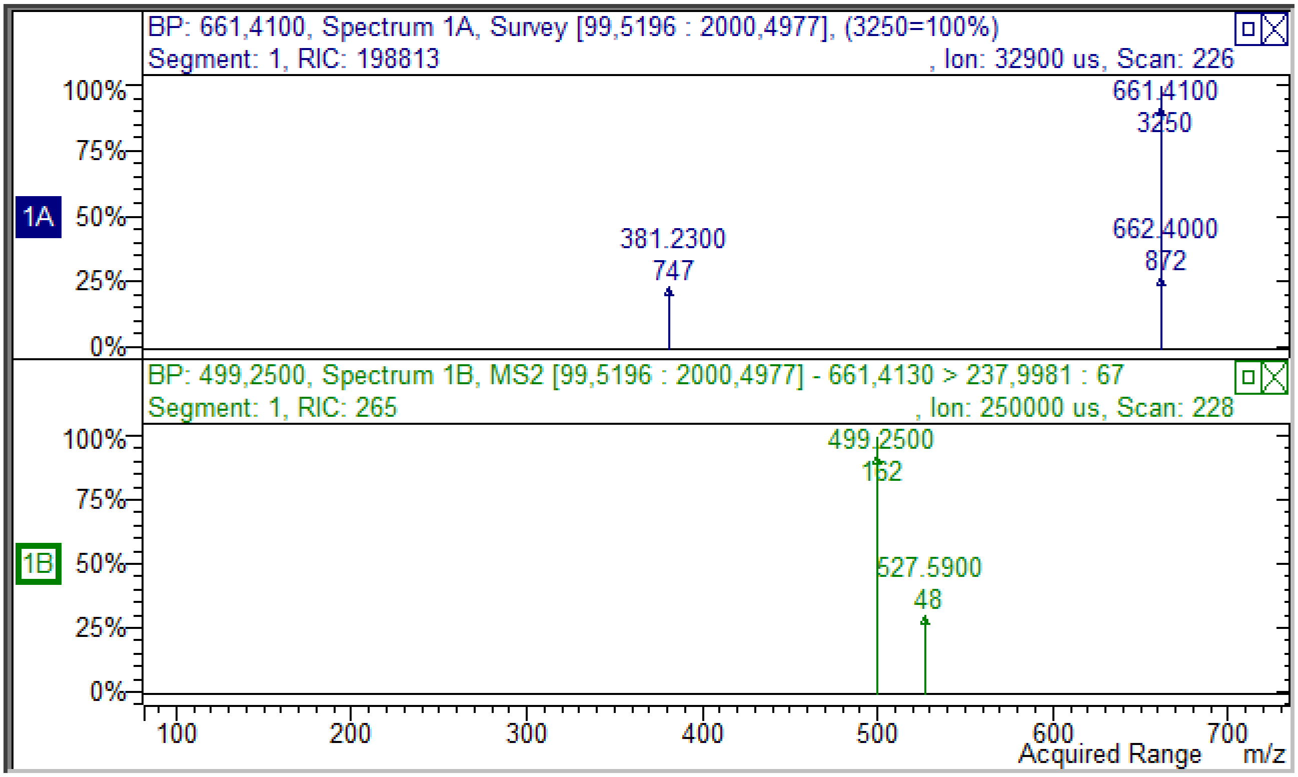

Methoxy piceatannol hexoside

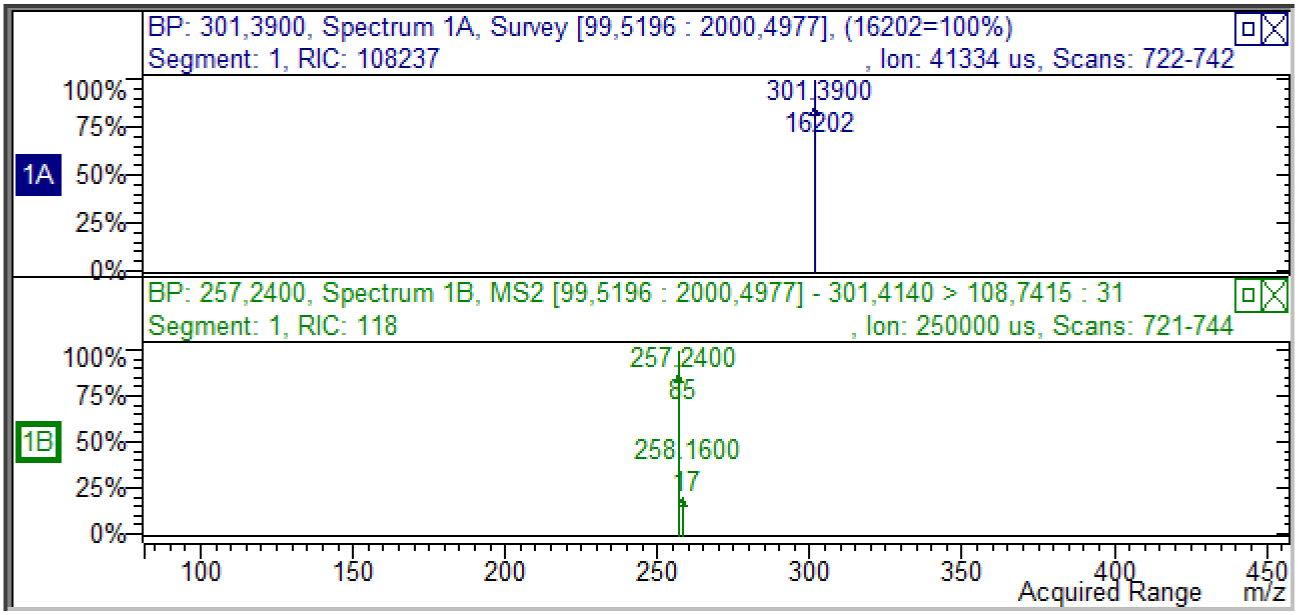

Abietic acid
